# Human Behavior Recognition Model Based on Feature and Classifier Selection

**DOI:** 10.3390/s21237791

**Published:** 2021-11-23

**Authors:** Ge Gao, Zhixin Li, Zhan Huan, Ying Chen, Jiuzhen Liang, Bangwen Zhou, Chenhui Dong

**Affiliations:** 1 School of Computer Science and Artificial Intelligence, Aliyun School of Big Data, School of Software, Changzhou University, Changzhou 213000, China; gaoge19kaoyan@163.com (G.G.); jzliang@cczu.edu.cn (J.L.); z2636735448@163.com (B.Z.); 2 School of Microelectronics and Control Engineering, Changzhou University, Changzhou 213000, China; lizhixin2021@cczu.edu.cn (Z.L.); chenying8787@cczu.edu.cn (Y.C.); d13303459683@163.com (C.D.)

**Keywords:** human activity recognition, wearable sensor, sliding window segmentation, frequency-domain characteristics, classifier selection

## Abstract

With the rapid development of the computer and sensor field, inertial sensor data have been widely used in human activity recognition. At present, most relevant studies divide human activities into basic actions and transitional actions, in which basic actions are classified by unified features, while transitional actions usually use context information to determine the category. For the existing single method that cannot well realize human activity recognition, this paper proposes a human activity classification and recognition model based on smartphone inertial sensor data. The model fully considers the feature differences of different properties of actions, uses a fixed sliding window to segment the human activity data of inertial sensors with different attributes and, finally, extracts the features and recognizes them on different classifiers. The experimental results show that dynamic and transitional actions could obtain the best recognition performance on support vector machines, while static actions could obtain better classification effects on ensemble classifiers; as for feature selection, the frequency-domain feature used in dynamic action had a high recognition rate, up to 99.35%. When time-domain features were used for static and transitional actions, higher recognition rates were obtained, 98.40% and 91.98%, respectively.

## 1. Introduction

Human activity recognition (HAR) is to recognize the actions performed by a person through a series of observations of the person and their surroundings. To improve user experience, HAR has been widely used in medical care, social security, behavior analysis, traffic safety and other industries. In the field of health care, HAR has been successfully applied to behavior monitoring [1], observation [2] and classification [3] of the elderly, medical diagnosis of patients [4], rehabilitation and physical therapy [5]. In the field of social security, the detection of abnormal people [6] and the tracking of suspicious objects [7] are inseparable from HAR technology, which improves the precision attack ability against criminals [8]. In the behavioral analysis industry, HAR is used to monitor and identify family behavior [8]. In the field of traffic safety [9], the detection of driver fatigue is helpful to prevent traffic accidents caused by drowsiness during driving.

Human activity classification methods mainly include vision system-based technology and wearable sensor system-based technology. Vision system-based technology appeared earlier than wearable sensor-based technology. In the past few decades, many effective research methods have been proposed. Its core processing stages mainly include data preprocessing, object segmentation, feature extraction and classifier implementation [10]. However, vision-based HAR still faces many challenges. On the one hand, video data is more likely to cause privacy disclosure, image processing occupies a lot of storage space and calculation involves considerable costs. On the other hand, the position and offset angle of the observer, the subject’s body size, clothes, background color and light intensity all affect the accuracy [11]. Compared with the method based on vision technology, the method based on inertial sensors often has lower costs, as well as stronger robustness and portability in various environments [12]. At present, many smartphones and smart wearable devices have been equipped with multiple sensors (such as accelerometers and gyroscopes) and the sensor data are easier to obtain. However, the change in installation position also changes the performance of identification. Therefore, in the research study [13], the authors explored the role of sensor position in the design of an HAR system in order to optimize its effect. With these advantages, human activity recognition based on wearable sensors has attracted more and more attention in recent years.

The wearable sensor system can measure several human physiological parameters and send data to the cloud platform with the help of smartphones. The cloud system can perform multiple tasks, such as data cleaning, data storage and data analysis. Under the electronic health monitoring system, doctors can see the patient’s data in real-time, especially for disease monitoring, prevention and treatment [14] to make a diagnosis. For example, abnormal gait and hand tremors are detected by analyzing human motion. Reference [15] designed and developed an electronic health monitoring prototype. Both wired and wireless communications can connect sensors to devices. Serial communication can be used to import the sensor data into the local server, or the wireless network or GPRS module can be used to send the data to the cloud medical server. Hosseinzadeh et al. [16] proposed a health monitoring system based on the Internet of Things to monitor life signals and identify biological changes in the elderly. Attaoui et al. [17] proposed an ECG telemedicine based on wearable sensors, which combines neural networks and the Internet of Things. The authors of [18] briefly described that the occurrence of accidental falls in the elderly leads to death. Studies around the world have also made many attempts and developed various sensors to predict the risk of falls in the elderly. Reference [19] systematically summarized the application of wearable sensors in these tasks.

At present, the main challenge to deal with the HAR problem is still the problem of motion characteristics. Time-domain features [20] are widely used as basic features in behavior recognition systems [21], but different types of actions are different and the classification effect of a single feature on different actions may be different. The characteristics of the time domain, frequency domain and time–frequency domain for HAR are discussed in detail in reference [22]. After feature extraction, how to select a machine learning algorithm to realize behavior classification and recognition is also a core problem to be considered. At present, there are two algorithms [12] used to study the HAR problem, the deep algorithm and the shallow algorithm. The HAR model based on deep learning can automatically extract salient features through different filters for recognition [23]. The deep algorithm is a black box model, has no inherent easy explanation, needs a large number of datasets for training and has a high computational cost. Due to these limitations, the shallow machine learning method is still preferred when the training dataset is very small or fast training is required.

Another challenging problem is the recognition of switching movements. Because the incidence of posture switching is low and the duration is shorter than other basic physical activities [24], most previous human behavior recognition systems ignored posture switching. However, in many practical situations, users can perform multiple tasks in a short time [25], such as a fitness or disability monitoring system. At this time, it is very important to determine posture conversion. In fact, in the case of human behavior recognition system and posture transition perception, the classification changes slightly and the lack of specified posture transition may lead to poor system performance [26].

This study compared the effects of three groups of features (including time-domain, frequency-domain and time–frequency-domain combined features) on the classification of various actions and then quantitatively analyzed the recognition performance of different actions under different feature subsets and learning classifiers.

The main contributions are as follows:

(1) According to the different dynamic characteristics of various actions, a classification and recognition model of human daily actions and switching actions is proposed. The model adopts different features to classify different actions, which avoids the problem that the unified features cannot represent multi-class action information and reduces the number of features involved in classification.

(2) The oversampling technology was used to eliminate the influence of unbalanced datasets and then transition actions were classified and identified as basic actions to improve the flexibility of this method in practical applications. Experiments compared the recognition performance of different actions under different feature subsets and multiple classifiers. The results show that the classification effect of transition actions and dynamic actions on support vector machines was the best, while the classification effect of static actions on ensemble classifiers was the best.

The rest of the paper is arranged as follows: Section 2, related works; Section 3, classification framework; Section 4, experimental results and analyses; Section 5, conclusions.

## 2. Related Work

HAR machine learning methods for smartphones equipped with inertial sensors can be divided into shallow algorithms (such as support vector machine (SVM), decision tree (DT), random forest (RF), K-nearest neighbor (KNN)) and deep algorithms. The main difference between a shallow algorithm and a deep algorithm lies in the way of feature extraction, that is, manual extraction or automatic extraction [27]. In addition, most wearable sensors are still limited by hardware resources and cannot quickly access the activity recognition results that need a lot of calculation. Therefore, to make HAR more suitable for wearable devices with limited hardware resources, most of the traditional analysis methods based on deep learning theory are abandoned by researchers [28], who continue to choose shallow machine learning methods. Bayat et al. [29] used the data of smartphones placed in their pockets and hands to recognize human activities and compared the performance of different classifiers. The experiments showed that the in-hand data used the voting combination of Multi-layer perceptron (MLP), LogitBoost and SVM classifier and the overall classification accuracy was 91.15%, while the in-pocket data used the voting combination of MLP, LogitBoost and simple logic and the classification accuracy was 90.34%. Ha and Ryu [30] proposed an ensemble method called Error Correction Output Coding (ECOC), with the random forest as the basic learner and the classification accuracy was 97.8%. Bhuiyan et al. [31] used a multi-class support vector machine (MC-SVM) as a classifier to classify five kinds of daily actions performed by humans and achieved good recognition performance. Minarno [32] compared the classification performance of DT, RF, KNN, logistic regression (LR), SVM and ensemble voting classifier (ECLF). The accuracy of the logistic regression method was 98% and the accuracy of the support vector machine classification method was 93.86%.

At present, the extracted features in the HAR system are divided into three categories: time feature, frequency feature and time–frequency feature [33]. When using time–frequency-domain features, Saha et al. [34] found that the data characteristics of accelerometer and gyroscope sensors were the best in the integrated classifier, with an overall accuracy of 94%. The data analyzed by Mohamed et al. [35] were the combination of accelerometer data from arms, belts and pockets; their classification recognition rate reached 98.9% on random forest. Ronao and Cho [36] found that the overall accuracy was improved to 93.18% by using the two-stage continuous hidden Markov model (TS-CHMM). Kastner et al. [37] used Generalized Learning Vector Quantization (GLVQ) for classification and the accuracy reached 96.23%. Using only time-domain features, a study conducted by Sufyan et al. [38] found that the best accuracy rate of each basic activity classification was given based on the classification of MLP and NBtree. The results obtained by Daghistani and Alsharmari [39] showed that the best overall classification accuracy rate using Adaboost (j48) achieved 94.034%. Gupta and Kumar [40] found that the best overall accuracy rate of the study on human sitting, standing, walking and running activities by the Adaboost classifier was 98.83%. Using the frequency-domain and time–frequency characteristics, Jiang et al. [41] obtained 97.59% of the total accuracy by using a deep convolution neural network.

For studies that focus on long-term activity monitoring, ignoring transition activities has little impact on the results, but, for the recognition of short-term actions, transition actions cannot be ignored. A lot of work on transition activities regard transition activities as a new activity similar to basic activities. Thien Huynh et al. [42] used SVM as a classifier to identify transition activities, obtaining a superior machine learning model. However, in real scenarios, transition activities can be easily classified as other activities, resulting in poor system performance.

At present, most of the existing works aim to extract unified features for actions, which has great drawbacks. Unified features should take into account multiple actions, which leads to an increase in the number of features. On the contrary, when the number of features is reduced, the classification effect is reduced. Most of the existing works aim to select classifiers empirically and there is no comparison of classifiers for action classification. At the same time, there are relatively few studies on transition movement. To solve the above problems, this paper divided the actions, compared the extracted features on different classifiers and analyzed the features and models that were more conducive to action classification.

## 3. Classification Framework

**Figure 1:**
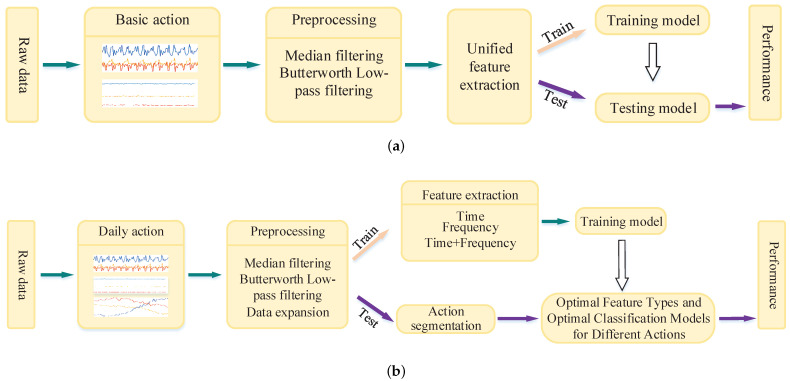
Schemes follow the same formatting. If there are multiple panels, they should be listed as (**a**) traditional HAR recognition model and (**b**) multi-feature and multi-classifier action recognition model.

Traditional methods to extract features for basic actions are shown in Figure 1a; our model is shown in Figure 1b.

### 3.1. Multi-Feature and Multi-Classifier Action Recognition Model

The action recognition model of multi-features and -classifiers proposed in this paper is shown in Figure 1b. The following improvements were made on the traditional method:

(1) In addition to basic actions, we considered transition actions as basic action recognition.

(2) Since the number of transition samples was much smaller than the basic action, we balanced the dataset.

(3) As the action data came from a continuous sequence of complex actions, first, the basic action was segmented. The action segmentation method derives from our team’s previous research results [43]. The introduction of this method is shown in Section 4.3.1 below.

(4) Compared with the traditional method, which only uses a single time-domain and frequency-domain feature for classification, we fully considered the behavior characteristics of different actions, reasonably divided the action types and extracted different types of features for different actions for model training.

(5) According to the features extracted from different actions, the training model obtained the optimal feature type and the optimal classifier and the results were directly used in the test set to obtain the optimal classification results.

The actions of the whole dataset were divided into two subsets; 70% of the data were selected to generate training data and the rest were selected to test. Then, the training set was used to train the model and the generated training model was used for the prediction of the test set. The features extracted from the training model are shown in Table 1. Finally, we used 10-fold cross-validation [44] and performance evaluation indicators to measure the generalization and accuracy of the model.

### 3.2. Data Preprocessing

Generally, the sensor data collected in the real scene inevitably contain noise. The median filter with core 5 and butterworth low-pass filter with a cut-off frequency of 20 Hz is usually used for noise reduction. Because 99% of its energy is contained below 15 Hz [45], this rate is sufficient to capture the movement of the human body.

**Figure 2:**
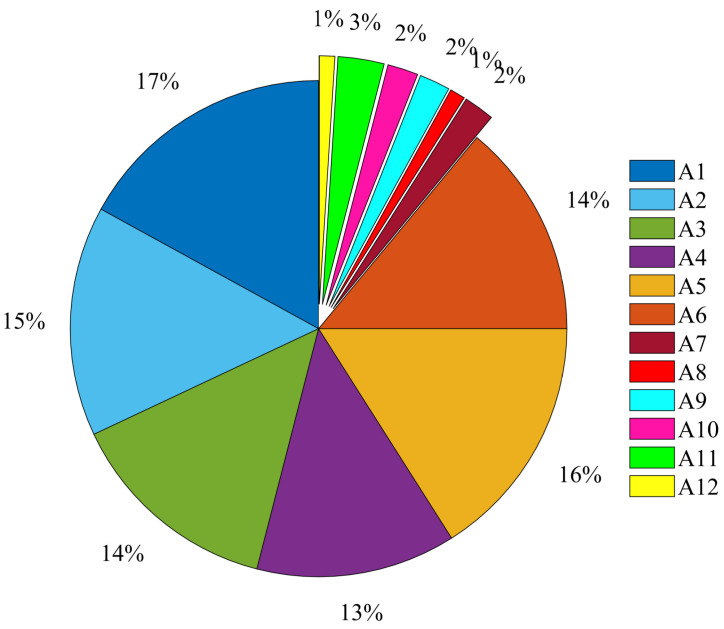
Percentage of different action categories in dataset.

According to the actions described in the dataset and the duration of each action, the average duration of transition action was 3.7 s, while the basic activity was about 20.1 s. The signals collected by a volunteer were extracted and the data of 12 actions (6 basic actions and 6 transition actions) were statistically analyzed (see Figure 2). The specific action categories are represented by A1–A12 in Table 2.

Transition activities are different from basic daily activities. The duration of transition activities is very short, the length of duration is not fixed and the sample size of transition actions is smaller than that of other basic actions. Therefore, to classify and identify the transition action as the basic action, it is necessary to oversample the transition data or under-sample the basic action data. It is pointed out that the oversampling method leads to an overfitting problem, while the under-sampling method leads to the loss of key information.

The SMOTE technology, proposed in reference [46], is widely used. It oversamples a few classes by creating synthetic examples. This method makes the constructed classifier include a larger decision area of nearby minority classes and improves the accuracy of minority class classification. Considering the short duration of transition activities, some transition activities are less than one window in size. The sample size meeting the size of a window is also small, which is not enough to provide the information to generate a better training model. Therefore, we also used the SMOTE algorithm to oversample a few classes for data expansion.

### 3.3. Feature Extraction and Model Training

To make the extracted features more suitable for the types of actions, different features were selected for training for different action training sets. At the same time, the number of single category samples of transition actions was expanded, which was roughly consistent with the number of single category samples of basic actions. Therefore, we could divide the original data into three categories: dynamic, static, and transition and extract features.

**Figure 3:**
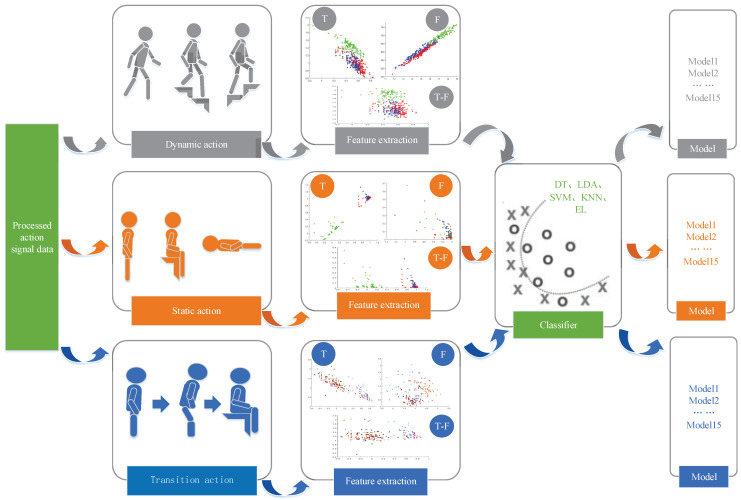
Feature extraction and classifier training of the model.

The specific features in Figure 1b were extracted and the classifier model training details were carried out (see Figure 3). In this paper, the divided basic actions and transition actions were divided in more detail. The divided actions were divided in extract features and trained models on five alternative classifiers; each property feature generated five models. Finally, a total of 15 training models were obtained for each type of action under three features and five classifiers (in the figure, time represents time-domain features, frequency represents frequency-domain features and time + frequency represents the combination of time–frequency-domain features).

#### 3.3.1. Feature Extraction

Six new sets of data (including each axis signal of acceleration and angular velocity) were obtained by deriving the original six-axis data. Secondly, the Euclidean norm was obtained from the original acceleration and angular velocity data to obtain two new groups of data. Therefore, a total of 14 sets of data were obtained. The sliding window has a good recognition effect on a single action, which effectively avoids the problem that the extracted features cannot well represent the real characteristics of the data due to different action lengths. To solve this problem, this paper applied an overlapping time window to slice the filtered data according to the window length. In our work, the length of each window was set to 2.56 s and the overlap was set to 50%, intercepting the action signal. Nine frequency-domain features and five time-domain features were extracted for each of the 14 groups of data in each window, so that 126 frequency-domain features and 70 time-domain features could be obtained for each sliding window. The time-domain and frequency-domain features were extracted by each window in the paper (see Table 1).

**Table 1:** Time-domain and frequency-domain features extracted from each window.

	Feature
Time	Max, Min, Range, Mean, Standard deviation
Frequency [47]	Center of gravity frequency, Mean square frequency, Root mean square frequency, Frequency variance, Frequency standard deviation, Mean value, Standard deviation, Median, Peak

#### 3.3.2. Classifier

The type of action, the number of features and the quality of raw data affect the effect of the classifier. On the premise of determining the training set and features, the selection of a classifier directly affects the effect of the whole classification. In machine learning, classifiers are divided into linear and nonlinear categories according to model parameters and input characteristics. The linear classifier has good interpretability and low computational complexity; the disadvantage is that the fitting effect of the model is relatively weak. The nonlinear classifier has a strong fitting ability; the shortcomings are insufficient data, easy overfitting, high computational complexity and poor interpretability. Based on the advantages and disadvantages of the above classifiers, this paper selected five classifiers for model training, namely, linear discriminant analysis (LDA) and KNN among the linear classifiers and DT [48], SVM [49] and ensemble learning (EL) [50] among the nonlinear classifiers.

## 4. Experimental Results and Analyses

### 4.1. Data Description

The experimental data of this paper were obtained from the dataset of “human activity recognition using smartphones” in the UCI (UC Irvine, University of California, Irvine) machine learning repository, which is publicly available [51]. For this dataset, smartphones were installed at the waist of 30 volunteers ranging in age from 19 to 48. The smartphones generated six common human activity data and transition movement data. A transition action is the transition from one stable state to another and the change between them is usually strong; this steady-state behavior is defined as basic activity. In this paper, the basic actions were further classified. To generate data, the smartphone used two sensors, an accelerometer and a gyroscope. The accelerometer measured the subject’s triaxial linear acceleration and the gyroscope measured the subject’s triaxial angular velocity at a constant rate of 50 Hz. These activities were manually marked with the help of recorded video. Although these tasks were carried out under laboratory conditions, volunteers were asked to perform a series of activities freely to be closer to the activities collected from natural datasets; Table 2 shows the activities carried out for this dataset and the division of execution actions made in this paper. Each action executed is represented by a combination of letters and numbers.

### 4.2. Experimental Setup and Performance Measurement Criteria

The experimental platform was mounted on Intel (R) Core (TM) i5-6200u@2.30 GHz on a Dell computer; the operating system was Windows 10 and MATLAB programming language tools were used. For activity recognition, indicators such as accuracy, recall, accuracy, F1 score, ROC curve and AUC value were intensively applied. It is proved that these evaluation methods are feasible.

To evaluate the performance of the model, this paper used four evaluation indexes: accuracy, accuracy, recall and F1 score [52].

### 4.3. Comparison of Experimental Results

#### 4.3.1. Division of Basic Movements

##### Reasons for Basic Action Division

Simple actions with long duration and repeatability are collectively referred to as human basic actions, as shown in Table 2. To identify basic actions with different dynamic characteristics, it is necessary to extract a large number of features, which not only causes information redundancy, but also brings a large amount of calculation to the computer, making the time of model training relatively long. In the experimental method (method 3) in this section, 126 frequency-domain features proposed in this paper were selected to classify 6 types of actions and the effect of the SVM classifier was improved compared with the literature [32]. The constructed feature vector was also trained and tested on other classifier packages—DT, RF, KNN, LR and ECLF classifiers. The comparison among the recognition rate obtained and those reported in reference [32] is shown in Table 3.

The overall recognition rate of six types of actions is reported in Table 3. The authors of [53] used 248 features and the accuracy of several depth learning methods was lower than that of traditional methods. This study selected the same experimental setting as that in the literature [32]. The authors of [32] did not carry out feature selection and directly used 561 time-domain and frequency-domain features extracted during data acquisition. In this paper, only 126 frequency-domain features were selected and the recognition rate of the SVM classifier was about 3% higher than that obtained in [32]. The reason why the effect on SVM was the best is that the dataset was nonlinear and separable. Therefore, when the parameters were correctly selected, the nonlinear SVM using the RBF kernel function showed a better classification effect.

**Figure 4:**
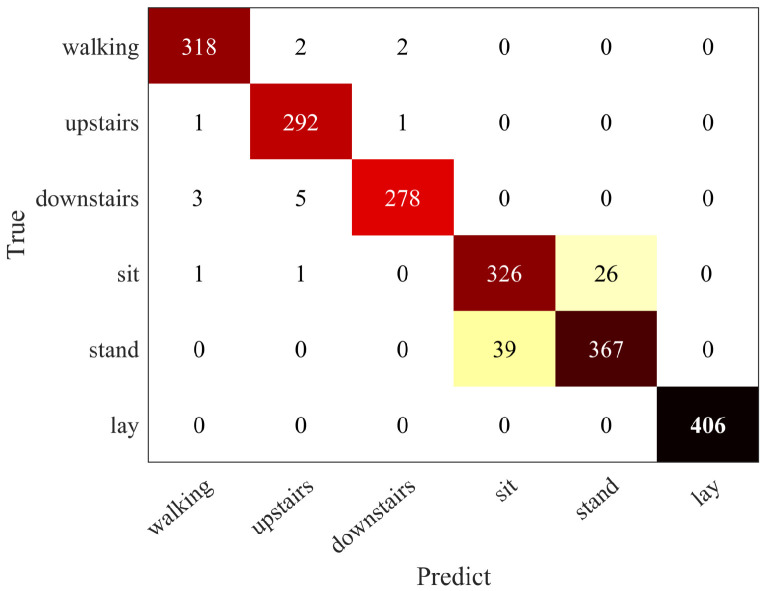
Basic action confusion matrix.

The classification effect of frequency-domain feature information of six basic actions on SVM is further given in Table 4. Activities A1, A2, A3 and A6 produced superior precision, ranging from 98.24% to 100.00%, while activities A4 and A5 had poor identification precision, 91.04% and 92.07%, respectively. The two poorly classified activities were “sitting” and “standing” in static actions (the confusion matrix is shown in Figure 4); the classification of “sitting” and “standing” is easy to be confused because the signal energy of human motion is concentrated in the low-frequency part. Compared with the periodicity and fluctuation amplitude of dynamic actions, static actions have no periodicity and small fluctuation. Their frequency-domain information does not have high separability, so more constraints are needed on the frequency-domain features for the recognition of static actions. Therefore, this paper considered subdividing the basic actions into dynamic and static actions for classification and processing, respectively (see Table 2).

##### Basic Action Segmentation Method

**Figure 5:**
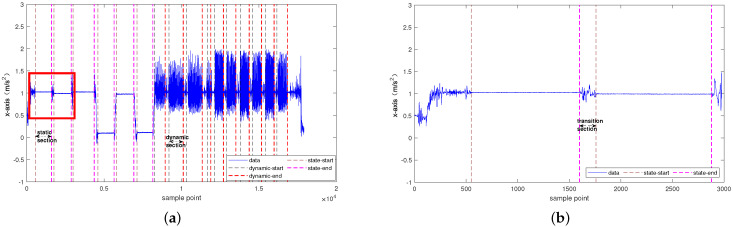
(**a**) Basic action segmentation sketch. (**b**) Traditional action segmentation sketch.

The segmentation of various actions is a key problem in this study. The action segmentation method adopts the previous research results of the team. The segmentation of basic actions is shown in Figure 5a. Based on the small fluctuation of static actions, the threshold approximation method was used to segment static actions. Secondly, the dynamic action had strong periodicity and the peak point detection method was used to segment the dynamic action. Finally, due to the continuity of human activities, the actions between static actions were identified as transition actions based on context analysis, as shown in the red framed portion of Figure 5a, which is enlarged as shown in Figure 5b. The three segmented actions were used for specific action recognition.

#### 4.3.2. Performance of Subdivision Action on Different Classifiers

**Figure 6:**
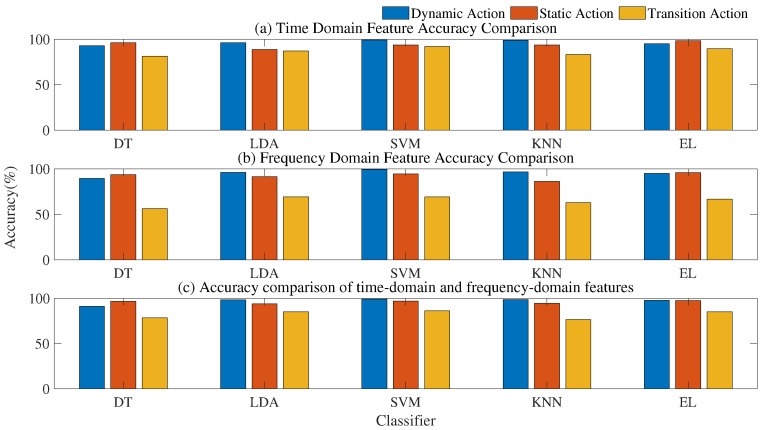
Accuracy comparison of the same features on different classifiers.

The experimental results in Section 4.3.1 show that the effect of action classification with different dynamic characteristics was also very different under the same classifier. Therefore, considering whether different actions are applicable and have a better recognition rate under different classifiers, this section classifies and recognizes the features extracted from subdivided actions on five different classifiers to find out the classifier with the best effect on action classification. Under the same number of features of the three types of actions, the results of the training set on different classifiers were compared (see Figure 6).

Whatever the characteristics, SVM was the best classifier to recognize dynamic actions, with a recognition accuracy of more than 99%. Transition actions were also classified best on SVM, with a recognition accuracy of 91.98%. DT was the worst classifier for the classification of two types of actions because DT is prone to overfitting and ignores the correlations among data (see Figure 6). Because the two kinds of action features were nonlinear separable, the key of nonlinear SVM was to map the linearly nonseparable samples in the input space to the linearly separable feature space. Therefore, the feature space mapped by the classifier had better linear separability and solved the problem of high-dimensional features. The generalization ability of the model was improved.

There was a big gap between static action and the other two kinds of action. This kind of action had the best classification effect in integrated learning, up to 98.40%. The bagging method in ensemble learning was used in this paper, which mainly reduced the variance and improved stability through repeated training. Ensemble learning classification uses the combination of multiple learners, which helps to reduce the risk of falling into the local optimal solution, to improve the overall generalization performance. In addition, the combination of multiple learners also helps to expand the hypothesis interval and may learn better approximation. Therefore, static actions achieved the best classification effect on this classifier.

**Figure 7:**
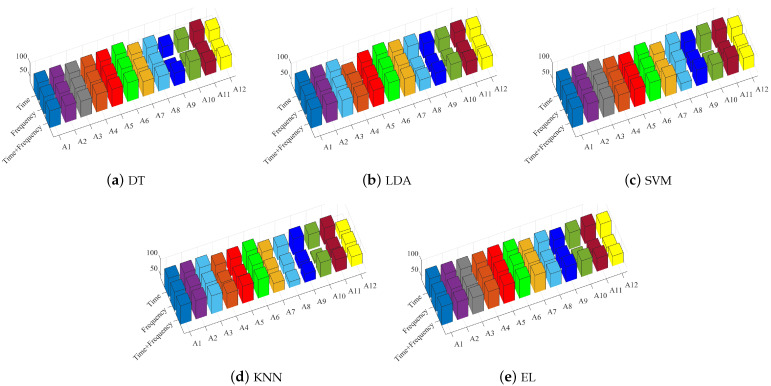
Action classification results under different classifiers. (**a**) Classification effect of action features on DT. (**b**) Classification effect of action features on LDA. (**c**) Classification effect of action features on SVM. (**d**) Classification effect of action features on KNN. (**e**) Classification effect of action features on EL.

The performance of specific 12 types of action features on different classifiers are shown Figure 7.

#### 4.3.3. Feature Evaluation of Single Action Based on Optimal Classifier

According to the analysis in Section 4.3.2, we know the best classifier for subdivision action classification. This section studies and gives the specific feature performance evaluation of various actions under the best classifier.

**Figure 8:**
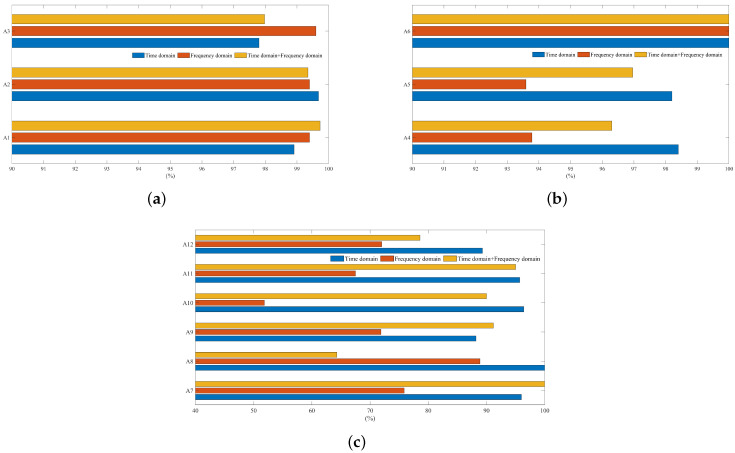
(**a**) Comparison of recall rates of dynamic actions under different characteristics. (**b**) Comparison of recall rates of static actions under different characteristics. (**c**) Comparison of recall rates of transition actions under different characteristics.

The superiority of the proposed method is reflected by comparing the previous research results. The proposed model was tested on the UCI dataset, so the selected comparative study was also carried out on the UCI dataset. A bar chart comparison of recall rate identified under different characteristics of three specific actions is shown in Figure 8. The comparison of accuracy and recall of each action category under the best features is represented in Table 5 and Table 6.

A3, the frequency-domain feature of single action, had the highest recognition rate. By comprehensively analyzing the overall accuracy and recall rate of single-action recognition, we can infer that the effect of frequency-domain features was the best (see Figure 8a) and this property feature achieved a good balance between different behaviors. It was also found that, although the signals of quasi-periodic actions with large fluctuation were concentrated in the low-frequency range, the frequency-domain information showed high separability. The frequency-domain feature performance evaluation is shown in Table 5. Single action reached a recall rate of more than 99.5%, in which the recognition precision of the A2 and A3 actions reached 100%. Compared with the literature [54], the recognition rate of these two kinds of actions was improved; especially, the recall rate of the A2 action was increased by about 4%.

The bar graph comparison of the recall rate of specific action recognition in a static action is reported in Figure 8a. The experimental results in Section 4.3.1 show that the recognition effect of static actions, especially “sitting” and “standing”, was poor. This is because the static action signal was the sensor signal recorded when the subject was still. The acceleration sensors are affected by gravity and there is an acceleration value in the vertical direction; the acceleration values in the other two directions vibrate up and down at zero. Compared with the steep and obvious dynamic action signal with large fluctuation, the static signal appears stable and with little fluctuation. The experiments show that the time-domain characteristics of static actions made the recall rate of three types of static actions the highest (see Figure 8b). The recall rate of the time-domain features of the three static actions shown in the figure is higher than that of the other two features. The precision rate using only the time-domain features of actions A4 and A5 was more than 98% (see Table 5). This is the time-domain information, which makes the feature distribution of the two types of static actions have obvious differences, thus increasing the recognition precision. Although the number of classification errors of the two specific static actions was greatly reduced under the time-domain characteristics, it did not solve the confusion of the two classifications. It was also found, from the bar graph, that the static action recognition rate decreased after the combination of time-domain features and frequency-domain features, which fully reflects the importance of feature engineering. The increase in the number of features may interfere with the useful features and then reduce the recognition precision. The experiment also found that the action of “lay” was special. No matter what characteristics were used for classification, the evaluation index of this kind of action was as high as 100%, which is higher than the 98.1% index in the literature. This is because the vertical axis of this kind of action is affected by gravity changes, which leads to the characteristic distribution being different from the other two kinds of action.

The classification recall rate of multiple transition actions under different features is shown in Figure 8c. The recall rate of any transition action using frequency-domain features was the lowest. The recall rate of time-domain features of the A8, A10, A11 and A12 transition actions was the highest and the recognition rate of the combination of time-domain and frequency-domain features of the other two actions was the highest. Comprehensively evaluating in combination with the histogram in Section 4.3.2, the overall recognition effect of only time-domain features was higher than the other two types of features. The specific effects of single action performance evaluation are shown in Table 5 and Table 6. It can be seen, from the table, that the recognition precision of the six transition actions was more than 90%, but the recall rate of A9 and A12 actions was low. This result shows that, among the action samples of this category, the number of original correct samples accounted for a high proportion of the number of identified correct samples and, for all correct samples, the number of predicted correct samples was small. According to the comparison table, the precision and recall rate of the method proposed in the article are much higher than those in the literature [54]. Compared with the results in [43], the recall rates of A7, A8 and A10 were greatly improved. Therefore, for the transition activities with short duration and violent fluctuation, measures such as data expansion and extracted time-domain features are beneficial to improve the recognition effect of transition actions.

Summarizing the above analysis (see Table 7), it was found that the frequency-domain feature has a better classification effect for dynamic actions, while the static actions and transition actions have a better classification effect under the time-domain feature. The factor considered here is that the frequency-domain feature better expresses the periodic information in the signal and the frequency-domain feature of quasi-periodic actions has high separability. However, the content of static action and transition action cycle is small and the resolution of frequency-domain information is not high.

## 5. Conclusions

In this study, based on the built-in sensor data of smartphones, human behavior was classified and identified. Firstly, the transitional actions between human continuous actions were balanced as samples and identified as basic actions. Secondly, the basic actions were divided more carefully and different characteristics were extracted from the processed transition actions; then, the classification and recognition were compared on multiple learning classifiers. The experiments show that the oversampling of transitional actions was conducive to action recognition. Secondly, the ensemble learning classifier had better classification performance for static action features and SVM showed better classification performance for dynamic action features with large fluctuations. In addition, dynamic actions could obtain better classification results under a small number of frequency-domain features and several common time-domain features of static actions and transition actions could obtain a good recognition rate, which shows the importance of feature engineering for action classification and the necessity of classification processing. The performance measures used to evaluate the classification techniques included accuracy, precision, recall and F1 score. At the same time, the classification and comparison of each type of action also included performance indicators such as precision, recall and F1 score. In the future, we will explore the applicability of this method on other datasets and will continue to explore other methods to improve the recognition performance of transition actions.

This paper only used the SMOTE method, as the balanced sample method is too traditional and single. In addition, there is a widespread phenomenon of class imbalance in natural datasets, which represents a great test for the sample balance method. In the future, a more systematic sample balance method will be adopted to further study the influence of the sample balance method on transition action recognition.

## Figures and Tables

**Table 2:** Human daily actions.

Traditional Classification	Actions	Actions Description	Our Classification
Basic action	A1	Walking	Dynamic action
	A2	Walking Upstairs	
	A3	Walking Downstairs	
	A4	Siting	Static action
	A5	Standing	
	A6	Laying	
Transitional action	A7	Stand-to-Sit	Transitional action
	A8	Sit-to-Stand	
	A9	Sit-to-Lie	
	A10	Lie-to-Sit	
	A11	Stand-to-Lie	
	A12	Lie-to-Stand	

**Table 3:** Comparison of classification accuracy for basic actions using different methods.

Reference	Classifier	Accuracy	Activities	Subject	Sensors	Features
Literature [53]	DCNN	94.18%	Sit, Stand, Lie, Walk, Walking Upstairs, Walking Downstairs	20	Three-axis accelerometer, gyroscope and magnetometer	248
	FRDCNN	95.27%				
Literature [32]	DT	93.44%	Sit, Stand, Lie, Walk, Walking Upstairs, Walking Downstairs	30	There-axis accelerometer and gyroscope	561
	RF	96.73%				
	KNN	96.21%				
	LR	98.40%				
	SVM	93.86%				
	ECLF	97.60%				
method3	DT	93%	Sit, Stand, Lie, Walk, Walking Upstairs, Walking Downstairs	30	There-axis accelerometer and gyroscope	**126**
	RF	96.13%				
	KNN	90%				
	LR	82%				
	**SVM**	**96.60%**				
	ECLF	97.18%				

**Table 4:** Performance evaluation of each activity based on SVM classifier.

Action	Precision	Recall	F1-Score
A1	99.70%	98.24%	98.96%
A2	98.79%	99.69%	99.24%
A3	98.34%	98.67%	98.50%
A4	91.81%	91.04%	91.42%
A5	91.04%	92.07%	91.55%
A6	100%	100%	100%

**Table 5:** Comparison of precision rate between the proposed method and other methods.

Precision (%)	A1	A2	A3	A4	A5	A6	A7	A8	A9	A10	A11	A12
AW-TD [54]	99.7	×	×	98.1	97.4	×	68.5	58.7	90.6	86.6	×	×
Literature [43]	99.0	100	96.6	98.6	98.8	99.3	100	100	89.6	100	77.9	100
Our method	**99.7**	100	**100**	98.1	98.6	**100**	96	100	**90.9**	92.6	**90.0**	96.2

**Table 6:** Comparison of recall rate between the proposed method and other methods.

Recall (%)	A1	A2	A3	A4	A5	A6	A7	A8	A9	A10	A11	A12
AW-TD [54]	96.3	×	×	90.6	99.2	×	89.2	86.0	92.9	89.2	×	×
Literature [43]	100	95.6	99.7	99.8	98.7	98.1	94.7	79.1	100	87.1	100	93.1
Our method	99.4	**99.4**	99.6	98.4	98.2	**100**	**96**	**100**	88.2	**96.4**	95.7	89.3

**Table 7:** Summary of this work.

Action	Best Features	Best Classifier
Dynamic action	Frequency-domain	SVM
Static action	Time-domain	EL
Transition action	Time-domain	SVM

## Data Availability

Not applicable.
